# Using theatre to address mental illness stigma: a knowledge translation study in bipolar disorder

**DOI:** 10.1186/2194-7511-2-1

**Published:** 2014-01-21

**Authors:** Erin E Michalak, James D Livingston, Victoria Maxwell, Rachelle Hole, Lisa D Hawke, Sagar V Parikh

**Affiliations:** Division of Mood Disorders, Department of Psychiatry, University of British Columbia, 2255 Wesbrook Mall, Vancouver, V6T 2A1 Canada; Saint Mary’s University, 923 Robie St, Halifax, Nova Scotia B3H 3C3 Canada; Crazy for Life Co., P.O. Box 1354, Sechelt, British Columbia V0N 3A0 Canada; School of Social Work, University of British Columbia, ARTS Bldg, 3333 University Way, Kelowna, British Columbia V1V 1V7 Canada; Université de Saint-Boniface, 200 Avenue de la Cathedrale, Winnipeg, MB R2H 0H7 Canada; University of Toronto, 399 Bathurst Street, Toronto, Ontario M5T 2S8 Canada; University Health Network, 190 Elizabeth Street, Toronto, Ontario M5G 2C4 Canada

**Keywords:** Bipolar disorder, Stigma, Theatre, Knowledge translation, Mixed methods, Narrative medicine

## Abstract

**Background:**

Reduction of the stigma of mental illness is an international priority; arts- and contact-based approaches represent a promising mode of intervention. This project was designed to explore the impact of a one-woman theatrical performance on attitudes towards bipolar disorder (BD) on people with BD and healthcare providers.

**Methods:**

A playwright and actress who lives with BD developed a stage performance - 'That’s Just Crazy Talk’ - targeting stigmatizing attitudes towards BD. Prospective, longitudinal and sequential mixed methods were used to assess the impact of the performance on people with BD (*n* = 80) and healthcare providers (*n* = 84). Qualitative interviews were conducted with 33 participants (14 people with BD and 19 healthcare providers).

**Results and Discussion:**

Quantitatively, healthcare providers showed significantly improved attitudes immediately post-performance, but this change was not maintained over time; people with BD showed little quantitative change. Qualitatively, both people with BD and BD healthcare providers showed enduring and broadly positive changes. A theatrical presentation designed to reduce stigma produced immediate impact on healthcare providers quantitatively and significant qualitative impact on people with BD and healthcare providers. Additionally, the utility of using mixed-method approaches in mental health research was demonstrated.

## Background

Mental health is determined by myriad factors, not simply the absence of specific symptoms or disorders. For people living with psychiatric conditions, stigma can play a pernicious role. Stigma is a dynamic, multifaceted social process in which people are devalued and discredited on the basis of a stereotyped social status or personal characteristic (Weiss et al. 2006). It has been consistently implicated as a key contributor to poor outcomes for many people who live with stigmatized health conditions, such as psychiatric conditions (Livingston and Boyd 2010). Although a large body of literature has accrued in relation to stigma and mental illnesses broadly (for review, see Hinshaw and Stier 2008;Schomerus et al. 2012), the literature on the specific relationship between stigma and bipolar disorder (BD) is comparatively lean. Nevertheless, this body of evidence does indicate that stigma experiences and perceptions are a common occurrence for people with BD and their families and that stigma can have severe repercussions (Michalak et al. 2011). For example, higher perceptions of stigma are associated with increased symptom severity and reduced functioning (Aydemir and Akkaya 2011;Cerit et al. 2011;Vázquez et al. 2011) and greater concealment, social withdrawal and social anxiety among people with BD (Aydemis and Akkaya 2011;Michalak et al. 2006;Michalak et al. 2007).

From a treatment perspective, people with higher self-stigma are less inclined to seek professional help (Yap et al. 2011), less likely to adhere to treatment when they do seek help (Fung et al. 2008;Tsang et al. 2010) and more likely to experience poorer therapeutic alliance with healthcare providers (Kondrat and Early 2010). Mental healthcare providers have been found to have relatively positive attitudes towards people with mental illness but are not free from stigmatizing attitudes and expectations, particularly with respect to the social acceptance of people with mental illness. Given the complex interrelations between stigma, symptoms, treatment seeking, and disorder management and treatment, modifying stigma may have major therapeutic benefit.

Research suggests that stigma reduction initiatives are more likely to be effective when tailored to the clinical profile of specific conditions (Hinshaw and Stier 2008;Sartorius and Schulze 2005), yet few stigma interventions targeted towards BD have been developed. Emerging evidence also indicates that an effective multifaceted strategy to prevent and reduce mental illness stigma would include creative arts and contact-based approaches (where contact approaches refer to anti-stigma interventions that rely on planned interactions between people with mental illness and the public (Corrigan 2012)). Theatre holds particular potential as one example of a creative arts-based approach, employing the tools of ethnography and artistry to convey complex messages with social and personal meaning. It also has entertainment value, which may facilitate dissemination. To date, the impact of theatre-based interventions have been applied in a variety of physical health conditions, such as brain injury and end-of-life care (Rossiter et al. 2008;Lorenz et al. 2004). Theatre has also been specifically employed to fight mental illness stigma. For example, the performance arts have been shown to help reduce stigma toward mental illness among teenagers and young adults (Faigin and Stein 2008;Roberts et al. 2007;Twardzicki 2008). One study evaluated a mental health festival, which brought tougher multiple arts-based interventions, and found modest positive impacts (Quinn et al. 2011). Theatrical interventions represent an example of narrative medicine (Greenhalgh and Hurwitz 1999) and a specific knowledge translation (KT) strategy (Eakin and Endicott 2006).

Here, we report on a study that investigated the effects of a theatrical performance that told a personal story about struggling with and managing stigma from the perspective of a woman living with BD. Our primary objective was to examine the effects of this intervention on the attitudes of people who live with the condition and healthcare providers.

## Methods

### Study design and participants

The study adopted a prospective, longitudinal, sequential mixed methods design in order to assess the impact of the performance on stigma using qualitative and quantitative approaches. Study participants included people with lived experience of BD and BD healthcare service providers in two large metropolitan Canadian cites: Vancouver and Toronto. Healthcare provider participants were aged 19 years or older, involved in the provision of care (direct or indirect) to people with BD and able to communicate in English. Participants in the people with BD group were aged 19 years or older, had a self-reported diagnosis of BD, and were able to communicate in English.

Ethical approval for the study was provided by the University of British Columbia and the University of Toronto, and written informed consent was obtained from all the participants. The participants were remunerated in the form of a $10 coffee card for participating in the quantitative evaluation, $30 for the qualitative evaluation and all participants received a DVD recording of the performance at the end of the study.

### Recruitment and study procedure

The study participants were recruited with multiple methods including our CREST.BD research network’s face-to-face, on-line and social media communications (e.g. public education events, email newsletter announcements, postings on the team’s website, Facebook and Twitter accounts). Study advertisements were disseminated through a wide range of consumer-focused (e.g. Mood Disorders Association of British Columbia, Mood Disorders Association of Ontario and Canadian Mental Health Association) and healthcare provider-focused associations (e.g. Canadian Psychiatric Association). Additional email announcements were sent through hospital and university email communications in Vancouver and Toronto.

People who were interested in attending a performance were asked to register by telephone or on line. At the point of registration, they were asked to self-select as a person with BD or a BD healthcare provider. Registration preference was given to research participants, but general audience members not involved in the research process were welcome at all performances, space permitting. In the week preceding the performance, potential research participants were emailed a consent form, background information and sociodemographic form, and two standardized stigma measures (described below). Research participants, therefore, completed the baseline (T1) stigma measures either in the 7 days leading up to the performance or on the actual day of the performance. The measures were re-administered immediately after the performance (T2), but prior to the question and answer period with the actress. In addition to the stigma measures, the T2 questionnaire included customized questions asking participants to evaluate various aspects of the performance.

Research participants who provided consent to re-contact were also requested (by email and telephone, up four times before being classified as a non-responder) to completed the stigma scales 3 to 4 months after the performance (T3).

Drawing on the strengths of a sequential mixed methods design, the qualitative methods were embedded within the overall design, and the findings of the quantitative analyses informed the development of the qualitative interviews (Creswell and Clark 2011;Klassen et. al 2012). The participants were purposively selected for a 3-month interview based on the nature of their pre-post change on the quantitative stigma measures, with participants selected from the subsamples of participants who demonstrated a statistically reliable reductions or increase in stigma on at least one of the measures and participants who experienced no reliable change. Interviews occurred at 3 months in order to allow for the assessment of whether any post-performance changes were maintained over the short to medium term and to allow time for the detection of any behavioural change.

Semi-structured interviews were conducted to elicit an in-depth conversation of the participants’ perceptions of the impact of the play in order to complement the quantitative findings. The interviews were conducted by telephone. Nineteen or 56% were performed by EM, an experienced qualitative researcher, and the remainder by one of two peer researchers (a counselling psychology student or a therapist, both of whom live with BD), as per our community-based participatory research (CBPR) approach. All interviews were digitally recorded and transcribed verbatim. Thematic analysis was used to identify themes across participant interviews (Braun and Clark 2006). Three members of the research team analyzed the first seven transcripts together to create a coding framework. Subsequent transcripts were co-analyzed and discussed in relation to the emerging coding framework and themes identified. Once analyzed, the quantitative findings were compared with the themes that emerged from the qualitative analysis. These interpretive processes provided a context for the trends found in the quantitative analyses: the qualitative findings helped explain in quantitative findings (Creswell and Clark 2011;Klassen et al. 2012).

### Intervention

This study represents a CBPR project in which academic researchers worked closely with an established actress and playwright who lives with BD type I (Victoria Maxwell, Crazy for Life Co., Sechelt, British Columbia, Canada) and developed a one-woman stage play specifically targeted towards BD stigma. VM and the research team worked closely during the early stages of developing the performance, for example, an early draft of the play was reviewed by the investigators to confirm that its themes mapped onto stigma issues identified in the literature. Specifically, the literature identifies internalized stigma experienced by the individual with BD and difficult encounters in workplace, relationship, and general settings (Michalak et al. 2011;Vázquez et al. 2011); all of these were captured in the play. After these initial discussions, VM was given full creative freedom.

In terms of producing the play, the services of a dramaturge who helped edit and shape the narrative arc of the storyline was engaged for the writing process. A director was hired for the rehearsal process prior to the premiere staging of the show. The rehearsal period was 6 weeks of 4-h meetings, 3 to 4 times each week. The research team was not present for any of the rehearsals. The final version of the play that premiered on stage was a result of an artistic process that came from the playwright’s personal experiences, unhampered by ideas or themes artificially imposed by the academic team. It should be noted that VM was not involved in carrying out the research or analyzing the data. The resulting performance - 'That’s Just Crazy Talk’ - consisted of an approximately 50-min performance, followed by an approximately 30-min question and answer period with the actress. The audiences comprised a mix of people with BD and BD healthcare providers and general audience members. The team was prepared to deal with any emotional decompensation of attendees at the performances, but no such crisis emerged.

In the performance, the dramatic narrative conveys the corollaries of decades of personal and familial mental illness, translating the narrator’s experiences of external and internal stigma into a vivid, often humorous and sometimes troubled, portrait of life lived with BD. Two performances were given in Vancouver and one in Toronto. All occurred in mid-sized, accessible and central downtown theatre venues and played to near-capacity houses.

### Measures

#### Day’s Mental Illness Stigma Scale

This self-report measure of stigmatizing attitudes toward mental illness consists of 28 items and contains seven subscales: interpersonal anxiety, relationship disruption, hygiene, visibility, treatability, professional efficacy and recovery (Day et al. 2007). In this study, we substituted the word 'mental illness’ with 'bipolar disorder’. For each item, the participants are asked to rate their level of agreement using a seven-point scale ranging from completely disagree (1) to completely agree (7). We also modified the scoring procedures by reverse-coding five items (1, 7, 9, 23 and 28) to ensure that higher scores were consistently indicative of greater levels of stigma across all items and subscales. The participants in both the people with BD and healthcare provider groups completed the Day’s Mental Illness Stigma Scale.

#### Mental Illness: Clinicians’ Attitudes Scale Version 4

This is a self-report measure of healthcare professionals’ attitudes toward people with mental illness (Kassam et al. 2010). The Mental Illness: Clinicians’ Attitudes Scale Version 4 (MICA-4) poses 16 statements for which the participants are asked to rate their level of agreement using an anchored six-point scale ranging from strongly agree (1) to strongly disagree (6). Reverse-coding was performed on ten items. The scores for each item were summed to produce a single overall score. Higher MICA-4 overall scores indicate greater levels of stigmatizing attitudes toward mental illness. Only participants in the healthcare provider group completed the MICA-4.

#### Internalized Stigma of Mental Illness scale

The Internalized Stigma of Mental Illness (ISMI) scale is a self-report questionnaire that is designed to measure the internalized, subjective experiences of stigma for people living with mental illness (Ritsher 2003). The ISMI consists of 29 items and contains five subscales: alienation, stereotype endorsement, discrimination experience, social withdrawal and stigma resistance. For each item, the participants are asked to rate their level of agreement using an anchored four-point scale that ranges from strongly disagree (1) to strongly agree (4). Items in the stigma resistance substance are reverse scored. The subscale scores and a total score are calculated by averaging the respondents’ ratings, with higher scores indicating higher levels of self-stigma. Only the participants in the people with BD group completed the ISMI scale.

#### Performance evaluation

The participants were asked a range of questions pertaining to their perceptions of the event such as whether they learnt something new during the performance, the teaching effectiveness of the performer, whether the performance had an emotional impact on them, whether they thought it could change public/mental healthcare providers’/their own acceptance of BD and overall satisfaction with the event. The evaluation forms were similar for people with BD and BD healthcare providers.

### Sample size and power

Power analysis with G*Power 3 indicated that a minimum of 71 participants in each group was needed for a power of 80% (*p* < 0.05, one-tailed, effect size = 0.30). Eighty-four people with BD and 87 healthcare providers consented to participate in the study. Data from four people with BD and three healthcare providers were omitted from the analysis because of missing data. The analyses were therefore performed on data from 80 people with BD and 84 healthcare providers. Three research participants met the study inclusion criteria as both a BD healthcare provider and a person with BD; for analytic purposes, these participants were included in the 'people with BD’ group for analysis. At T3, 37 (46.3%) people with BD and 43 (51.2%) healthcare providers were retained. Bivariate comparisons of participants who were or were not retained at T3 revealed no significant differences (*p* > 0.05) in gender, mean age and total mean scores on the DMISS, MICA and ISMI at T1.

### Data analysis

Quantitative data were analyzed using SPSS version 14 (for Windows, IBM Corporation, Armonk, NY, USA). Effect sizes were calculated using G*Power 3. Paired sample *t* tests were conducted in order to compare T1 and T2 subscale and total scores of the dependent measures among people with BD and healthcare providers. Pairwise deletion was used to remove specific missing values from the analysis. To assess the effect of time (T1, T2 and T3) on average total stigma scores, we performed a repeated measures MANOVA, with 'time’ specified as the within-subjects factor. Only total scores were used, rather than subscale scores, for analyzing the three waves of data in order to reduce the number of comparisons made given the small sample size at T3. The total scores from cases with too many missing items (e.g. more than three) were removed from the analyses. Mauchly’s Test of Sphericity was significant on the DMISS for both groups in addition to the ISMI for people with BD, indicating that sphericity cannot be assumed. In these instances, we relied on the Greenhouse-Geisser correction procedure.

## Results

### Participants

A total of 164 participants (80 people with BD and 84 healthcare providers) were included in the quantitative analyses. Overall, the majority of the total sample were female (*n* = 127, 77.9%), and the average age was 41 years (SD = 12.6, range = 21 to 74 years).

For the people with BD sample specifically, 71% were female, 26% male and 2% otherwise gendered with a mean age of 42.4 years (SD = 12.2, range = 21 to 71 years). In comparison, in the healthcare provider group specifically, 84% were female and 16% male with a mean age of 40.2 years (SD = 12.9, range = 23 to 74 years). The majority of the people with BD sample described themselves as white/Caucasian (*n* = 63 or 83%) with the second most commonly endorsed racial identity being Asian (*n* = 7 or 8.2%). On average, people with BD (*n* = 79) reported living with the condition for 10.1 (SD = 9.1) years. For the healthcare provider group, 16 (19.3%) of the sample were nurses, 8 (9.6%) psychologists, 6 (7.2%) psychiatrists, 6 (7.2%) occupational therapists, 5(6.0) psychology trainees, 4 (4.8) social workers and 38 (45%) coming from 'other’ healthcare disciplines. The average years of healthcare practice across all disciplines (*n* = 71) was 12.1 years (SD = 11.9). Three quarters of the healthcare provider sample (75.9%) had not seen a different performance by Victoria Maxwell, whereas 84% of the people with BD sample had not. No significant differences were observed in any of these descriptive variables between groups, or in distribution of healthcare provider versus people with BD participants across data collection sites (Vancouver vs. Toronto). In total, 195 non-research participants also saw the show across the three performances.

A total of 33 participants (9 males and 24 females) were included in the qualitative phase. The sample included 14 people with self-reported BD (BD type I = 8, BD type II = 5; NOS = 1) and 19 healthcare providers (2 psychiatrists, 4 nurses, 5 psychologists, 2 occupational therapists and 6 others). Of this sample, ten participants demonstrated significant change on the quantitative measures, demonstrating a reduction in stigma; 18 participants showed no change, and a further 5 demonstrated change for the worse.

### Quantitative results

#### General evaluation

In order to understand the 'face validity’ of the performance, questions on the play and its potential to alter stigma were asked: 98% of the participants described the show as 'good’ or 'excellent’ with positive feedback across people with BD, healthcare providers and general audience members. The performance was judged to have the potential to affect stigma, as 67% people with BD and 85% of healthcare providers thought the play could 'change public acceptance of BD’ (see Table 1). With the common DMISS, at baseline, healthcare providers had significantly lower total average scores than people with BD, *t* (162) = -4.90, *p* < 0.001, indicating that they held more positive attitudes toward people with BD (see Table 2).

**Table 1:** **Evaluation measures**

	Providers (***n*** = 84)		People w/ BD (***n*** = 80)		Test
	***N***	Valid% or mean	***N***	Valid% or mean	
Participant learned something new					
Poor (1)	0	0	2	2.7	
Fair (2)	0	0	2	2.7	
Neutral (3)	16	20.3	26	35.1	
Good (4)	42	53.2	26	35.1	
Excellent (5)	21	26.7	18	24.3	
M (SD)	79	4.06 (0.69)	74	3.76 (0.95)	*t* (132) = 2.27, *p* = 0.024^a^
Teaching effectiveness of presenter					
Poor (1)	0	0	0	0	
Fair (2)	1	1.3	0	0	
Neutral (3)	0	0	5	6.8	
Good (4)	11	13.9	17	23.0	
Excellent (5)	67	84.8	52	70.3	
M (SD)	79	4.82 (0.47)	74	4.64 (0.61)	*t* (138) = 2.12, *p* = 0.036^a^
Emotional impact on participant					
Poor (1)	0	0	0	*0*	
Fair (2)	0	0	3	4.2	
Neutral (3)	7	9.0	3	4.2	
Good (4)	20	25.6	28	38.9	
Excellent (5)	51	65.4	38	52.8	
M (SD)	78	4.56 (0.66)	72	4.40 (0.76)	*t* (148) = 1.39, *p* = 0.166
Length of event					
Poor (1)	0	0	1	1.4	
Fair (2)	1	1.3	1	1.4	
Neutral (3)	4	5.1	7	9.5	
Good (4)	21	26.6	31	41.9	
Excellent (5)	53	67.1	34	45.9	
M (SD)	79	4.59 (0.65)	74	4.30 (0.81)	*t* (151) = 2.52, *p* = 0.013
Overall satisfaction with content of event					
Poor (1)	0	0	1	1.4	
Fair (2)	0	0	1	1.4	
Neutral (3)	0	0	4	5.4	
Good (4)	20	25.3	19	25.7	
Excellent (5)	79	74.7	49	66.2	
M (SD)	79	4.75 (0.44)	74	4.54 (0.78)	*t* (113) = 2.00, *p* = 0.048^a^
Overall rating of event					
Poor (1)	0	0	1	1.4	
Fair (2)	0	0	0	0	
Neutral (3)	1	1.3	2	2.7	
Good (4)	19	24.1	21	28.4	
Excellent (5)	59	74.7	50	67.6	
M (SD)	79	4.73 (0.47)	74	4.61 (0.68)	*t* (129) = 1.33, *p* = 0.188^a^
Could event change public acceptance of BD					
No	0	0	1	1.9	
Somewhat	11	13.8	10	18.5	
Yes	69	86.3	43	79.6	
Could event change non-MH providers’ acceptance of BD					
No	0	0	na	na	
Somewhat	15	18.5	na	na	
Yes	65	81.3	na	na	
Could event change MH providers’ acceptance of BD					
No	1	1.3	na	na	
Somewhat	16	20.0	na	na	
Yes	63	78.8	na	na	
Did event change participants’ acceptance of BD					
No	24	30.4	20	37.0	
Somewhat	18	22.8	14	25.9	
Yes	37	46.8	20	37.0	

na, not applicable. ^a^Unequal variance *t* test.

**Table 2:** **Comparison of baseline levels of stigma (DMISS) between healthcare providers and people with BD**

	Provider			Person with BD					
	***N***	Mean	SD	***N***	Mean	SD	***t***	***df***	***P***
Stigma (DMISS) (*α* = 0.89)									
Treatability***	84	1.74	0.76	80	2.48	1.16	-4.82	135	0.000
Relationship disruption***	84	2.38	0.99	80	3.22	1.23	-4.82^a^	152	0.000
Hygiene**	84	1.93	0.99	80	2.44	1.30	-2.80^a^	148	0.006
Anxiety	84	1.71	0.89	80	1.97	0.99	-1.75	162	0.083
Visibility	84	3.40	0.84	80	3.57	0.80	-1.36	162	0.176
Recovery***	84	2.55	1.31	80	3.59	1.87	-4.10^a^	141	0.000
Professional efficacy	84	2.74	1.35	80	3.18	1.52	-1.95	162	0.053
Total***	84	2.26	0.63	80	2.79	0.74	-4.90	162	0.000

*p < 0.05, two-tailed; **p < 0.01, two-tailed;***p < 0.001, two-tailed. ^a^Unequal variance *t* test.

Tables 3 and 4 summarize the average scores on the stigma measures for healthcare providers and people with BD at T1 and T2. At T2, immediately following the intervention, healthcare providers demonstrated significant change across the five DMISS subscales and the total score, indicating improvements in attitudes toward BD. In comparison, the average scores for people with BD significantly changed on only one DMISS subscale (relationship disruption). The average DMISS total score for 42 healthcare providers who were interviewed at three time points was 2.29 (SD = 0.65) at T1, 2.07 (SD = 0.60) at T2, and 2.19 (SD = 0.60) at T3. A repeated measures MANOVA indicated that the time effect was significant for the DMISS total score, *F* (1.45, 59.33) = 5.28, *p* = 0.015; however, within-subjects contrasts revealed that the time trend was quadratic. Therefore, stigmatizing attitudes decreased immediately following the intervention but then increased to almost baseline levels at 3-month follow-up in providers. Average DMISS total scores for 36 people with BD who were interviewed at three time points were 2.73 (SD = 0.79) at T1, 2.70 (SD = 0.92) at T2, and 2.58 (SD = 0.86) at T3; the time effect was not significant, *F* (1.66, 57.95) = 1.72, *p* = 0.192.

**Table 3:** **Comparison of average stigma scores for healthcare providers (** ***n*** **= 84) before and after the performance**

Stigma measures	T1		T2		Repeated ***t*** test	***d***
	Mean	SD	Mean	SD		
DMISS						
Treatability	1.74	0.76	1.52	0.54	*t* (83) = 2.94, *p* < 0.004	0.32
Relationship	2.38	0.99	2.06	0.94	*t* (83) = 4.35, *p* < 0.001	0.48
Hygiene	1.93	0.99	1.67	0.87	*t* (83) = 3.42, *p* = 0.001	0.37
Anxiety	1.72	0.89	1.60	0.82	*t* (83) = 2.14, *p* = 0.035	0.24
Visibility	3.40	0.84	3.40	0.83	*t* (83) = 0.01, *p* = 0.990	0.00
Recovery	2.55	1.31	2.20	1.38	*t* (83) = 2.44, *p* = 0.017	0.27
Professional efficacy	2.74	1.35	2.53	1.32	*t* (83) = 1.78, *p* = 0.079	0.20
Total	2.26	0.63	2.07	0.61	*t* (83) = 5.55, *p* < 0.001	0.58
MICA						
Total sum	30.61	6.52	29.77	6.61	*t* (81) = 1.56, *p* = 0.123	0.17

DMISS, Day’s Mental Illness Stigma Scale; MICA, Mental Illness: Clinicians’ Attitudes Scale Version 4.

**Table 4:** **Comparison of average stigma scores for people with BD (** ***n*** **= 80) before and after the performance**

Stigma measures	T1		T2		Repeated ***t*** test (two-tailed)	***d***
	Mean	SD	Mean	SD		
DMISS						
Treatability	2.48	1.16	2.33	1.19	*t* (79) = 1.61, *p* =0.111	0.17
Relationship	3.22	1.23	3.00	1.22	*t* (79) = 2.42, *p* = 0.018	0.27
Hygiene	2.44	1.30	2.35	1.36	*t* (79) = 0.98, *p* = 0.332	0.11
Anxiety	1.97	0.99	1.97	0.99	*t* (79) = 0.03, *p* = 0.978	0.00
Visibility	3.57	0.80	3.68	0.85	*t* (79) = -1.10, *p* = 0.277	0.14
Recovery	3.59	1.87	3.48	1.95	*t* (79) = 0.69, *p* = 0.495	0.07
Professional efficacy	3.18	1.52	2.99	1.53	*t* (79) = 1.39, *p* = 0.170	0.15
Total	2.79	0.74	2.71	0.80	*t* (79) = 1.76, *p* = 0.083	0.19
ISMI						
Alienation	2.35	0.70	2.23	0.77	*t* (77) = 2.61, *p* = 0.011	0.31
Stereotype	1.55	0.45	1.56	0.45	*t* (76) = -0.48, *p* = 0.635	0.04
Discrimination	2.20	0.63	2.24	0.72	*t* (74) = -0.61, *p* = 0.542	0.08
Withdrawal	2.04	0.68	2.05	0.75	*t* (78) = -0.32, *p* = 0.751	0.02
Resistance	2.02	0.61	1.93	0.58	*t* (76) = 1.14, *p* = 0.259	0.14
Total	2.01	0.49	1.98	0.55	*t* (74) = 1.00, *p* = 0.319	0.13

DMISS, Day’s Mental Illness Stigma Scale; ISMI, Internalized Stigma of Mental Illness scale.

The results from the MICA-4 and ISMI revealed a different pattern. For healthcare providers, no significant change was seen between T1 and T2 on the MICA-4. Average MICA-4 total score for 40 healthcare providers who were interviewed at three time points was 29.40 (SD = 5.82) at T1, 27.58 (SD = 5.83) at T2, and 29.85 (SD = 6.47) at T3. A repeated measures MANOVA indicated a significant time effect for the MICA-4 total, *F* (2, 78) = 4.31, *p* = 0.017; however, within-subjects contrasts revealed that the time trend was quadratic. Therefore, stigmatizing attitudes decreased immediately following the intervention but then increased to baseline levels at 3-month follow-up. For individuals with BD, only one subscale of the ISMI (alienation) showed change from T1 to T2. The ISMI total scores remained stable between T1 and T2, *t* (74) = 1.00, *p* = 0.319. The average ISMI total scores for 34 people with BD who were interviewed at three time points were 2.04 (SD = 0.50) at T1, 2.02 (SD = 0.55) at T2, and 2.84 (SD = 0.36) at T3. A repeated measures MANOVA indicated a significant linear time effect, *F* (1.13, 37.20) = 30.24, *p* < 0.001. Therefore, stigmatizing attitudes were significantly elevated at 3-month follow-up for people with BD.

### Qualitative results

By and large, all individuals participating in the qualitative interviews indicted that the performance had a positive impact on their understandings of BD. In particular, they valued the unique learning opportunity (e.g. accessible presentation style) and the emotional impact of the performance. Specifically, they described how the performance informed them about the following: (1) the complexity and heterogeneity of BD experiences, (2) the possibility and opportunity for recovery (e.g. hope) and, relatedly, (3) a demystification of the experience of BD (see Table 5). The participants who had demonstrated significant positive change quantitatively typically described that this change occurred as a result of increased empathy, hope, and/or openness (for themselves and/or for others living with BD) (see Table 6). The subset of individuals interviewed who did not demonstrate significant change on the quantitative measures indicated that the performance had held meaningful impact for them; they described their experience of That’s Just Crazy Talk as a positive reminder and/or a confirmation of their views and beliefs regarding BD (see Table 7).

**Table 5:** **Qualitative findings across participants**

Qualitative finding	Participant quotes
Emotional impact of That’s Just Crazy Talk	It was downright entertaining and moving. 03-HP007
Authentic	Knowing that she’s an actress, but knowing that she actually lived it (…) had an impact. 03-HP009
Reliable	It just was kind of like reading your own journal but through someone else’s eyes. 02-HP006 (HP&CM)
Courageous	The key factors for me would be that she was really fearless about telling her story. 02-CM006
That’s Just Crazy Talk as a unique learning opportunity	I thought it was very helpful just to hear the experience from a person’s perspective who actually was struggling with the disorder. 02-HP010
The complexity and heterogeneity of BD experiences	More specifically that, I’m seeing that there’s more levels of disorder than what I’ve experienced. 01-CM029
The possibility and opportunity for recovery (e.g. hope)	That’s powerful, because it gives you an image that people can get better, you know. This is where she was at, and this is how she is now. 02-HP027
A demystification of the experiences of BD	I think it just reminded me that they’re just like you and me. They’re just a person who has a story. 02-HP006

CM = person with BD; HP = healthcare provider.

**Table 6:** **Subset of participants who demonstrated positive change (quantitatively)**

Qualitative finding	Participant quotes
Increased empathy	Going from less information to after the play having more information has (…) opened up my compassion. 01-CM029
1. Increased hope	Hope for recovery. (…) Anyone else that I meet that suffers from this and that is honest about what they’ve lived and how they’ve lived, and can share that – that gives me hope. 03-CM017
	It made me feel hopeful for the reduction in stigma. It made me feel hopeful for other people that were watching it that would be inspired by her. 01-HP43
2. Increased openness	I’m probably more vocal about how it, about talking about mental illness than I was before I saw it. 01-HP024
	It made me, you know, more comfortable and maybe, made me maybe a little more able to tell people I have bipolar disorder. 02-CM006

CM = person with lived experience of BD; HP = healthcare provider.

**Table 7:** **Subset of participants who did not demonstrate change (quantitatively)**

Qualitative finding	Participant quotes
Meaningful impact: a positive reminder and/or confirmation of their views and beliefs regarding BD	I think it did affirm the importance of being able to see things from the patient’s perspective and always keeping that in mind. 02-HP010

HP = healthcare provider.

## Discussion

Stigma is an important determinant of health and quality of life in people with mental illness (Hinshaw and Stier 2008;Schomerus et al. 2012) and has such has been prioritized as a key target by national bodies such as the Mental Health Commission of Canada (Kirby 2008). Bipolar disorder is certainly not immune to stigma; our own program of research has demonstrated that stigma is a major concern for people with BD and their family members (Michalak et al. 2011;Suto et al. 2012), as has that of other research groups (Mileva et al. 2013). Given its pervasiveness and insidious effects, stigma reduction interventions hold hope as a means of improving functioning, social support and health and quality of life in people with BD. Much work remains to be done, however, in terms of identifying effective stigma reduction interventions in mental illness broadly and in relation to BD specifically.

In this project, we utilized integrated KT methods to develop a new one-woman theatrical performance designed to ameliorate stigmatizing attitudes in two target groups: people with BD and BD healthcare providers. The intervention was deliberately designed to include both groups at the same event as an additional 'meta-message’ conveying the common humanity of both and modelling that the groups could mix in a non-clinical setting. At a broad level, we demonstrated high 'face validity’ for the intervention and observed high levels of satisfaction in both target groups, and the performance was clearly perceived as both engaging and entertaining. Our findings on the impact of the performance on stigmatizing attitudes were more complex and need to be unpacked in the context of the mixed methods research approach we utilized. In terms of the quantitative scores, in healthcare providers, we observed an immediate significant impact on one quantitative measure, with erosion of change over time. In people with BD, no significant change was apparent in quantitative scores with the exception of one subscale (the ISMI subscale that assesses feelings of alienation); in fact, a few participants appeared to get worse in terms of their stigma scores immediate post-performance.

Had we only assessed change in stigma via quantitative scales, these results would have suggested only a moderate short-term effect on healthcare providers and a small effect on persons with BD. However, we purposefully interviewed select participants several months after the performance in order to further evaluate the potential impact of the performance. A comparative and interpretive analysis of both quantitative and qualitative findings demonstrated that while quantitatively measured change in stigmatizing attitudes eroded over time, individuals participating in the qualitative interviews expressed continued positive effects from the intervention. For example, individuals participating in the qualitative phase described knowledge that had been acquired from attending the play (e.g., understanding about the spectrum of BD and the heterogeneity of experiences). In addition, both individuals with BD and healthcare providers expressed that the performance resonated with their experiences and had made them 'more hopeful’ , 'more empathetic’ and 'more open’.

Notably, the qualitative findings added important insights around the lack of quantitative change observed in some participants. When probed, these participants described what they interpreted as a floor effect, specifically, that they felt they already had a lot of empathy for individuals living with BD and a lot of knowledge about the condition. Interestingly, for these individuals, the notion of 'no change’ did not equate to 'no impact’. In fact, these participants described an impact where the play acted as a reminder and confirmation of their beliefs and attitudes, for example, participants described that the play reminded them of the importance of being empathetic and compassionate and of valuing the subjective nature of human experience. Indeed, arts-based approaches may represent a particularly effective method for reaffirming empathy and raising awareness about how people subjectively experience illness and health challenges.

Why did the quantitative change observed in the healthcare provider group erode over time? Other research studies have similarly sown relatively modest impacts from short-term interventions (e.g. Quinn et al. 2011;Faigin and Stein 2008); stigmatizing attitudes can be obstinate, consequently current recommendations call for continuous, long-term stigma-reduction campaigns rather than single, short-term interventions (Stuart et al. 2012). Extensive, long-term stigma-reduction campaigns will require substantial toolkits of interventions to maintain novelty and momentum. While each component of such toolkit may have a modest impact, multiple interventions may each reinforce the message of the other, together accomplishing broad-based, sustainable change (Rossiter et al. 2008). In support of this approach, we filmed one of the live performances of That’s Just Crazy Talk and produced a DVD version of the show, which was also demonstrated to have a positive impact on stigmatizing attitudes in healthcare providers (Hawke et al. 2013).

Is the impact of this performance specifically effective for people with BD or specialist BD healthcare providers? On the one hand, stigma is a social process that likely has universal features and consequences regardless of the specific disorder or social status. We focused on assessing the impact of That’s Just Crazy Talk on people with BD and healthcare providers. Although general audience members responded positively to the performance, we did not assess its impact on stigmatizing attitudes in this group. On the other hand, there is also evidence that different illnesses can be associated with different stereotypes (Crisp et al. 2000;Angermeyer et al. 2003;Durand-Zaleski 2012), leading to the suggestion that that anti-stigma initiatives will be more successful if they target a specific condition rather than mental illness broadly (Reavley and Jorm 2011).

### Design considerations

Several potential limitations to this research require consideration. The first grouping of limitations relates to our study sample. No confirmation of diagnosis of BD was made in the study; it is probable that a proportion of the lived experience sample may not have met standard psychiatric diagnostic criteria for BD. Further, the sample of participants with BD and healthcare providers was self-selected; it cannot be assumed that they represent the average person with BD or the average healthcare provider, and our attrition rate by T3 was substantial. Finally, no assessment of mood state was made in the participants with BD at the time of the performance.

The second grouping of limitations relates to study design. Our design did not include a non-intervention comparison group. There should also be consideration as to the timing of our assessment points. We purposefully conducted the T2 assessment point immediately after the performance but prior to the Q&A session, thinking that we wanted to assess the impact of the play specifically and that the Q&A session would not be standardized in delivery across different performances. However, this decision meant that we did not capture the impact of the post-performance discussions quantitatively, potentially a lost opportunity given that several participants mentioned in the qualitative interviews that this part of the intervention was particularly meaningful or evocative. A significant limitation in our study design occurs in that the 'intervention’ being evaluated at the T2 assessment point does not include the Q&A period. However, for T3, the Q&A is included in the 'intervention’ as participants were exposed to this between T2 and T3. The factors make it difficult to ascertain whether the impact was the result of the performance itself or the Q&A, although we will be addressing this point in the full report on the qualitative findings from the study.

There exists a growing body of research about using live performances to illustrate patients’ perspectives on illness for mixed audiences of healthcare providers and patients (e.g. Shapiro and Hunt 2003). Our research and qualitative questions were not, however, designed to capture any impact (positive or negative) of targeting both people with BD and healthcare providers simultaneously. Relatedly, perhaps the T2 assessment point was not ideally timed in terms of allowing participants adequate time to process what was, for some, emotionally moving material. With hindsight, it would have been interesting to assess the impact of the performance a day or two after the show.

Our third grouping of limitations relates to our selected assessment scales. A growing number of scales designed to measure stigma have emerged; however, their ability to detect change has not been fully tested. This is certainly true of the measures used in the current study. Moreover, the scales used in this study are explicit measures of attitudes and beliefs (i.e. conscious and controllable), which are prone to social desirability bias. The possibility of social desirability bias when completing stigma assessments should also be a consideration in research of this nature, perhaps by incorporating implicit measures of stigma, although the likelihood of this occurring would have been lessened as the questionnaires were completed anonymously.

## Conclusions

Theatrical traditions clearly hold the potential to impact audience members, both at affective and cognitive levels, and to foster insight and deepened understanding. In recognition of this, there is a growing body of literature on the use of drama to share health information across a diverse range of health conditions (Rossiter et al. 2008). Comparatively, little research has yet specifically focused on the use of theatre to impact mental illness stigma; this research project, to the best of our knowledge, represents the first to address stigma specifically in relation to BD. We demonstrated immediate impact on stigma in health care providers and enduring qualitative impact on stigma on individuals with BD through a theatre-based intervention. The power of arts-based approaches, which are consonant with the current emphasis on narrative-based medicine, may lie in their potential to reach and speak to an audience that may not be responsive to conventional methods for addressing stigma and may represent a yet-to-be fully tapped mechanism for change.
